# Nerve-Shattered Pensioners: More "Country Hosts" Needed

**Published:** 1918-02-09

**Authors:** 

**Affiliations:** Chairman, Country Host Institution.; Vice-Chairman.


*To the Editor of* The Hospital.

### Nerve-shattered Pensioners: More "Country Hosts" Needed.

*To the Editor of* The Hospital.

Sir,—The "Country Host" scheme came into being as the result of a letter from Dr. Thomas Lumsden published in the press six months ago. This scheme has been experimentally sanctioned and tried by the Ministry of Pensions and the London War Pensions Committee. All the patients who have resided with "Country Hosts" have made excellent progress and have put on weight at the average rate of roughly one pound per week, in spite of adverse weather and of the fact that all of them had been discharged from hospital because no more could be done for them.

The Ministry of Pensions is being asked by the London Committee and by the War Pensions Joint Advisory Committee for Kent, Surrey, and Sussex to extend to more pensioners the benefit of the scheme.

The aim of the "Country Host" scheme is by degrees to get these discharged soldiers back to useful life and to prevent their drifting into chronic invalidism, and the method adopted is to send them to work on the land or in gardens, under the care and supervision of "Country Hosts." More "hosts" within easy reach westwards of London are much needed. The following are the working conditions of the scheme:—

(1) The host provides free lodging, lights, firing, and attendance—*i.e.*, cooking for the man and care of his room—but incurs no medical responsibility for him or his well-being. (2) The host must have a good-sized garden or farm in which suitable light outdoor work can be found for the patient. (3) The men selected for treatment shall be of good character, sober, not suffering from any serious symptoms, and able to look after themselves. In case of ordinary illness the man should be attended by the local panel doctor. (4) An allowance of at least 15s. per week will, if required, be paid direct to the actual host—*e.g.*, the gardener housing the man. (5) Full instructions as to diet, rest, work, and general treatment will be sent to his host with each man. (6) The host is requested to report to the Hon. Organiser, on the forms supplied, every fortnight the progress of his guest. (7) The treatment will generally occupy three months, and towards the end of their course the men should be able to perform much useful agricultural work, thus increasing the country's food supply while at the same time confirming their own cure.

There must be very many people living quietly in the country away from the air-raid area who are anxious to help those who have suffered in the stress of battle. Here, then, is their opportunity in a practical form. There can be no more patriotic and dutiful service than that of helping back to health and happiness those whose nerves have been shattered in facing our foes and fighting for our freedom. Everyone willing to become a "host" or desiring further information, should communicate at once with the Hon. Secretary, Country Host Institution, 13 South Eaton Place, S.W. 1.

Sligo, Chairman, Country Host Institution.
Knutsford, Vice-Chairman.

